# Exploring the Role of Skull Base Anatomy in Surgical Approach Selection and Endocrinological Outcomes in Craniopharyngiomas

**DOI:** 10.3390/jcm15020896

**Published:** 2026-01-22

**Authors:** Alessandro Tozzi, Giorgio Fiore, Elisa Sala, Giulio Andrea Bertani, Stefano Borsa, Ilaria Carnicelli, Emanuele Ferrante, Giulia Platania, Giovanna Mantovani, Marco Locatelli

**Affiliations:** 1 Department of Clinical Sciences and Community Health, University of Milan, 20122 Milan, Italy; alessandro.tozzi@unimi.it (A.T.); ilaria.carnicelli@unimi.it (I.C.); giovanna.mantovani@unimi.it (G.M.); marco.locatelli@unimi.it (M.L.); 2 Department of Neurosurgery, Fondazione IRCCS Cá Granda Ospedale Maggiore Policlinico, 20122 Milan, Italy; giorgiofiore.doc@gmail.com (G.F.); stefano.borsa@policlinico.mi.it (S.B.); 3 Endocrinology Unit, Fondazione IRCCS Cá Granda Ospedale Maggiore Policlinico, 20122 Milan, Italy; elisa.sala@policlinico.mi.it (E.S.); emanuele.ferrante@policlinico.mi.it (E.F.); 4 Department of Neuroradiology, Fondazione IRCCS Cá Granda Ospedale Maggiore Policlinico, 20122 Milan, Italy; giulia.platania@policlinico.mi.it

**Keywords:** craniopharyngiomas, skull base surgery, hypothalamic syndrome

## Abstract

**Background/Objectives**: Craniopharyngiomas (CPs) are rare, generally benign tumors predominantly located in the sellar and suprasellar regions, associated with significant morbidity and complex surgical management. Despite high overall survival rates, patients frequently experience complications including visual impairment, pituitary dysfunction, diabetes insipidus (DI), and hypothalamic syndrome. Among these, hypothalamic obesity (HO) represents one of the most clinically challenging sequelae, often occurring early, lacking standardized medical treatment, and leading to substantial comorbidity and reduced quality of life. This study reports a single-center experience focusing on the relationship between skull base anatomy, surgical approach selection, and endocrinological outcomes. **Methods**: A retrospective analysis was conducted on patients diagnosed with CPs who underwent surgery by a dedicated team at our Department from January 2014 to January 2024. The approaches used were endoscopic (ER) and transcranial (TR). Preoperative imaging (volumetric MRI and CT scans) was analyzed using 3DSlicer (open-source software) for anatomical modeling of the tumor and skull base. Clinical outcomes were evaluated through follow-up assessments by a team of neuroendocrinologists. Data on BMI changes, DI onset, and hypopituitarism were collected. Statistical analyses consisted of descriptive comparisons and exploratory regression models. **Results**: Of 18 patients reviewed, 14 met the inclusion criteria. Larger sphenoid sinus volumes were associated with selection of an endoscopic endonasal approach (*p* = 0.0351; AUC = 0.875). In ER cases, the osteotomy area was directly related to tumor volume, independent of other anatomical parameters. Postoperatively, a significant increase in BMI (22.39 vs. 26.65 kg/m^2^; *p* = 0.0049) and in the incidence of DI (three vs. nine cases; *p*-value 0.0272) was observed. No clear differential association between surgical approach and endocrinological outcomes emerged in this cohort. **Conclusions**: Quantitative assessment of skull base anatomy using 3D modeling may support surgical approach selection in patients with craniopharyngiomas, particularly in identifying anatomical settings favorable to endoscopic endonasal surgery. Endocrinological outcomes appeared more closely related to tumor characteristics and hypothalamic involvement than to the surgical route itself. These findings support the role of individualized, anatomy-informed surgical planning within a multidisciplinary framework.

## 1. Introduction

Craniopharyngiomas (CPs) are rare, histologically benign tumors arising in the sellar and suprasellar regions, typically involving three regions of the hypothalamic-pituitary axis: the tuber cinereum, pituitary stalk, and pituitary gland [1,2]. Their incidence exhibits a bimodal distribution, with peaks in children and adults [1,2,3]. Histologically, CPs are classified into two main subtypes: adamantinomatous and papillary [3,4]. Surgical resection remains the treatment of choice, with reported 20-year overall survival rates of 87–95% in mixed adult and pediatric cohorts [5,6].

Despite their benign nature, CPs are associated with substantial recurrence rates and poor postoperative quality of life (QoL) [2,7], making them a clinical challenge [2,8]. Recent studies have focused on identifying preoperative predictors and intraoperative factors associated with reduced endocrinological and neurological morbidity, such as visual impairment, pituitary dysfunction, diabetes insipidus (DI), and hypothalamic obesity (HO) [2,3,9,10]. Key determinants of the extent of resection, recurrence risk, and postoperative hypothalamic dysfunction include tumor size and its relationship to the hypothalamus, whereas the surgical approach itself appears to play a more limited role [2,3,9,10].

Traditionally, CPs have been managed through transcranial routes (TRs). However, with the widespread adoption of endoscopic techniques, endoscopic transnasal resection (ER) has become increasingly utilized, with endoscopy in skull base surgery contributing to better outcomes [11,12]. This study aims to identify anatomical factors, derived from patient-specific 3D models, that may influence surgical approach selection. Additionally, we sought to explore the relationship between surgical route and long-term endocrinological outcomes.

## 2. Materials and Methods

### 2.1. Patients and Study Design

We retrospectively reviewed patients with CPs who underwent surgery at Fondazione IRCCS Ca’ Granda, Ospedale Maggiore Policlinico, University of Milan, Italy, between January 2014 and January 2024. Inclusion criteria were histological diagnosis of CPs (both papillary and adamantinomatous subtypes), availability of pre- and postoperative contrast-enhanced 3D T1-weighted MRI and preoperative volumetric CT imaging, and clinical data with a minimum follow-up of one year.

Data collected included demographic information (age and sex), clinical presentation, surgical approach, tumor histology, anatomical skull base parameters, resection outcomes, and rates of recurrence.

Given the retrospective design, the Ethics Committee waived study registration and the requirement for written informed consent for participation. Reporting followed the STROBE guidelines for cohort studies.

### 2.2. Data Collection

#### 2.2.1. Surgical Approaches

Surgical resection was performed by a dedicated multidisciplinary team led by the senior author. Two main approaches were employed:

- Endoscopic resection (ER), performed via a transnasal-sphenoidal corridor using a four-hand technique, including anterior sphenoidotomy and partial posterior nasal septectomy. Extended transsphenoidal approaches were employed when needed. Tumor removal involved the use of ring curettes, surgical aspirators, and sharp dissection. The osteo-dural defect was repaired using the 3F technique [13].
- Transcranial resection (TR) through a pterional craniotomy.

The choice of approach was individualized based on detailed preoperative anatomical assessment, tumor location and extent, and surgical expertise; these selection criteria are further discussed in the Section 4.

Data on pre-resection surgical procedures, such as third ventriculostomy (IIIVCS) or external ventricular drains (EVD), were also collected.

#### 2.2.2. Imaging Study

As a part of standard preoperative care, all patients underwent volumetric brain MRI and CT imaging for surgical planning, as follows:

- MRI imaging: Using a 3-Tesla Philips Achieva scanner (Philips Healthcare, Best, The Netherlands), 3D T1w (before and after gadolinium injection), FLAIR and T2w images were acquired.
- CT imaging: Acquired using a General Electric OPTIMA 660 scanner (GE Healthcare, Chicago, IL, USA), including a volumetric acquisition with bone window reconstruction.

Within 48 h after surgery, all patients underwent brain CT imaging to rule out surgical complications. Three months after surgery, an MRI was performed to identify residual pathology. Cases with radiologically evident residual tumor were classified as subtotal resections. Long-term follow-up included annual brain MRIs to monitor for tumor recurrence or progression. The time to progression or recurrence was measured in months from surgery date.

### 2.3. Outcomes

#### 2.3.1. Volumetric Analysis

**Figure 1:**
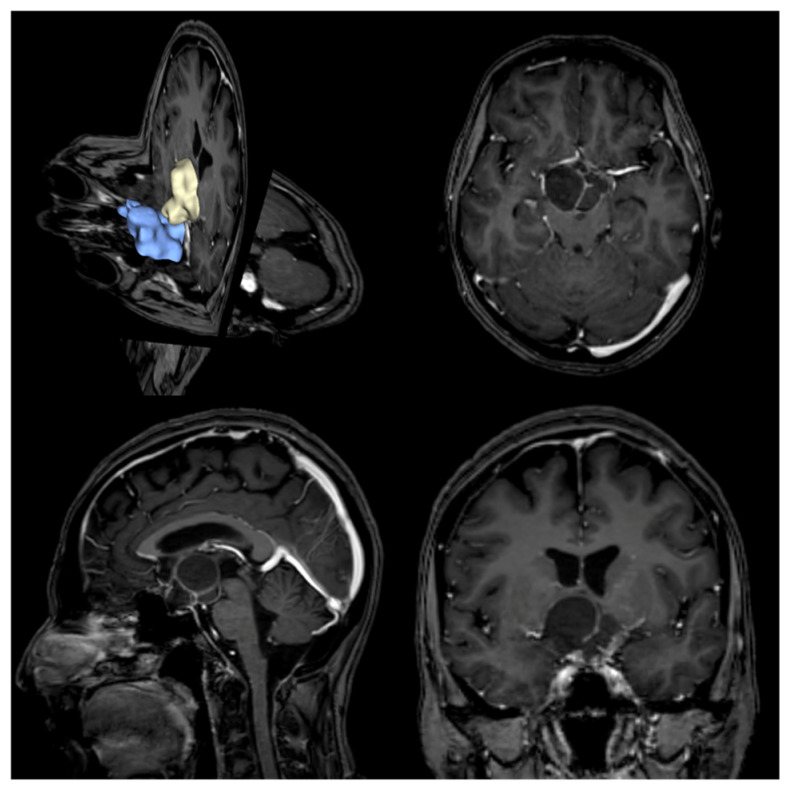
Case no 10: An adamantinomatous CP. In blue the sphenoidal sinus is represented, while in light yellow the tumor is represented.

**Figure 2:**
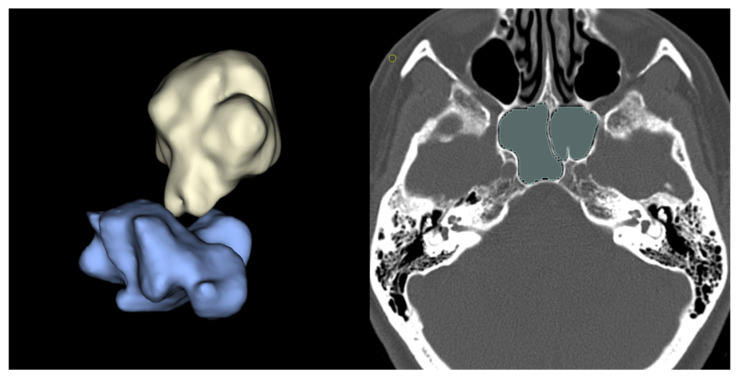
On the (**left**), a 3D representation of the sphenoidal sinus (light blue) and tumor (yellow). On the (**right**), the segmentation (green) of the sphenoidal sinus on CT scan (bone window).

Three-dimensional models were generated from DICOM imaging data. Preoperative MR and CT scans were processed using 3D Slicer software (version 5.8.0 for macOS) Tumors (from T1-weighted MR images) and sphenoid sinuses (from bone-windowed CT scans) were manually segmented, with individual volumes calculated in cubic millimeters. Each segmentation was converted to a 3D object and exported in STL format. Segmentations were performed by an experienced neuroradiologist (G.P.). Additionally, the intercavernous carotid distance (ICD) was measured in millimeters at the narrowest point between intracavernous carotid siphons using coronal multiplanar reconstructions on MR T1w sequences (Figure 1 and Figure 2) [14]. For ER cases, the osteotomy area (in square millimeters) was approximated by simplifying the shape to a rectangle. The area was calculated using the maximum cranio-caudal and latero-lateral diameters measured on the sellar floor plane, obtained from MPR of postoperative CT scans. All measurements and 3D model segmentations were performed blinded to clinical outcomes and surgical approach.

#### 2.3.2. Endocrinological Analysis

All clinical and metabolic follow-up was conducted by experienced neuroendocrinologists at our institution. Pituitary function was assessed both at diagnosis and after surgery. The first postoperative evaluation was scheduled for all patients one month after the surgical procedure, with subsequent hormonal assessments tailored to each patient’s specific needs. Follow-up duration was variable among patients; in general, all patients undergo lifelong surveillance, although the timing of evaluations differs on an individual basis. For the purpose of this study, postoperative data were recorded from the last available follow-up. Hypopituitarism was defined as the presence of one or more hormonal deficits. Hypoadrenalism was defined by basal cortisol level < 100 nmol/L or an abnormal response of cortisol in a dynamic test [15]. In particular, before June 2016 we considered inadequate a peak of cortisol 500 nmol/L, during 1 μg corticotrophin stimulation test (ACTH 1 mcg) or insulin tolerance test (ITT); after that time the diagnostic cut-off associated with the assay used at our institution (Roche 2) was 351 nmol/L [16,17]. A free thyroxine (fT4) under reference range in combination with not adequately increased TSH concentration was indicative of TSH deficiency [18]. Central hypogonadism was suggested in premenopausal women with low estradiol and low LH/FSH in conjunction with oligomenorrhea or amenorrhea, and in postmenopausal women in the presence of FSH levels inappropriately low for menopausal status. In men, central hypogonadism was defined by testosterone under reference range for age and normal/low LH/FSH in combination with clinical signs and symptoms [19]. Normal prolactin values were considered to be in the range of 2 to 20 mg/mL, with differences between sexes [20].

For diabetes insipidus, a urinary output > 40 ml/kg/day was considered an initial screening criterion [20]. The diagnosis was corroborated by the presence of hypernatremia and/or the requirement for desmopressin therapy. Only patients with diabetes insipidus at the time of hospital discharge were considered affected by DI in the early postoperative evaluation, whereas transient postoperative polyuria resolving before discharge was not classified as DI.

All hormonal deficits were appropriately treated with hormonal replacement therapy. BMI was calculated as body weight divided by squared height (kg/m^2^), both at diagnosis and at the last follow-up available [21]. Values of BMI ≥ 30 kg/m^2^ were considered suggestive of first-grade obesity, ≥25 and ≤29.9 kg/m^2^ suggestive of overweight [21]. Hypothalamic obesity was defined as postoperative weight gain resulting in obesity, defined by a BMI ≥ 30 kg/m^2^, documented in the medical record and occurring after surgery [22].

#### 2.3.3. Statistical Analysis

Given the observational design, regression analyses were planned as exploratory and hypothesis-generating. Variables of interest were reported and compared as follows:

- Frequencies were reported as a percentage and compared by chi-squared and Fisher exact tests according to sample size.
- The continuous normally distributed variables were reported as mean and compared through Student’s *t*-test or variance analyses.
- The continuous skewed distributed variables were reported as median and compared using the Mann–Whitney U-test.

Exploratory logistic regression models were used to examine associations between radiological and anatomical features, surgical approach selection, and endocrinological outcomes. The relevant explanatory variables were chosen based on the available literature as well as the statistical significance of the univariate analysis. Statistical significance was reached for *p*-values less than 0.05. A ROC analysis was performed for the models, using the area under the curve (AUC) as the performance metric.

All statistical analyses were performed using JMP Pro (version 15, SAS Institute Inc., Cary, NC, USA, 1989–2023) and R studio software (version 4.2.2, R Foundation for Statistical Computing, Vienna, Austria).

## 3. Results

Between January 2014 and January 2024, 18 patients with CPs underwent surgical resection at our department; 14 were included in the final analysis, while 4 were excluded due to incomplete data. A summary of the patient characteristics, clinical findings, and outcomes is provided in Table 1, Table 2 and Table 3.

Female patients were slightly more prevalent (female-to-male ratio 4:3), with a median age of 43 years (range 16–67) (Table 1). The mean follow-up was 60 ± 72 months (range 12–218, Table 1). Endoscopic resections were more prevalent than transcranial approaches (2:1), with 11 cases being primary surgical interventions. Three patients underwent cyst puncture combined with the placement of a subcutaneous reservoir (Rickham) via stereotactic or endoscopic techniques (cases no. 1, 2, 12). Postoperative complications included cerebrospinal fluid (CSF) leaks in 21.3% of cases (cases 4, 8, 14) and infections in 14.29% of cases (cases 11 and 13). All patients with CSF leaks underwent dural flap reconstruction via the endoscopic route as previously described [23]. One patient (case 2) experienced postoperative hemorrhage, requiring initial external and subsequent peritoneal ventricular shunting. No deaths were reported during the follow-up period. Residual tumor was detected in three patients (21.43%) who underwent adjuvant radiosurgery (cases 4, 5, 7). Tumor recurrence occurred in one patient (case 5), who had undergone STR via the transcranial approach (Table 2).

### 3.1. Volumetric Study

Radiological and anatomical features are summarized in Table 2. According to the Kassam classification [24], a high prevalence of high-grade tumors was observed, with types 3a/3b representing 71.43% of cases. The mean tumor volume was 7768 mm^3^ (1047–21,345), while the mean sphenoid sinus volume was 10,509 mm^3^ (5424–14,519). The mean intercarotid distance was 14.46 mm (11.42–18.58). For the ER subgroup, the mean osteotomy area was 102.15 mm^2^ (76.48–127.59).

**Figure 3:**
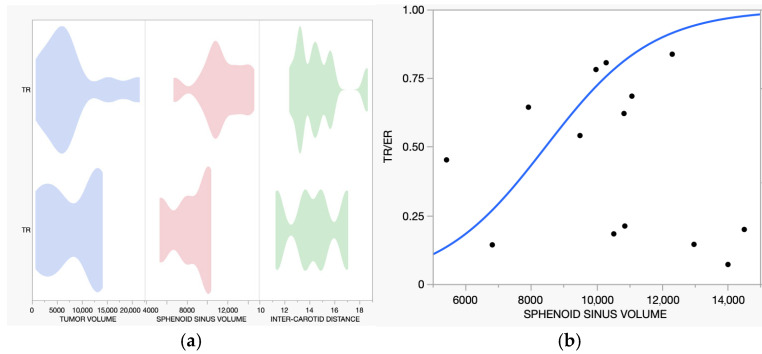
(**a**). The violin graph on the left displays the values of tumor volume (BLUE), sphenoid sinus volume (RED), and inter-carotid distance (GREEN), expressed in mm^3^ and mm, differentiated into the two groups ER and TR. No statistical difference was found regarding tumor volume and inter-carotid distance in relation to the surgical approach. (**b**). Patients who underwent ER presented a higher volume of the sphenoid sinus (black dot) at the time of surgery. The model (blue line) used had an R-square of 0.265 with a *p*-value of 0.0351, while the ROC curve showed an AUC of 0.875.

Linear regression analysis demonstrated that larger sphenoid sinus volumes were associated with the selection of ER (R^2^ = 0.265, *p* = 0.0351). The Receiver Operating Characteristic (ROC) curve demonstrated good discriminatory ability, with an area under the curve (AUC) of 0.875 (Figure 3).

**Figure 4:**
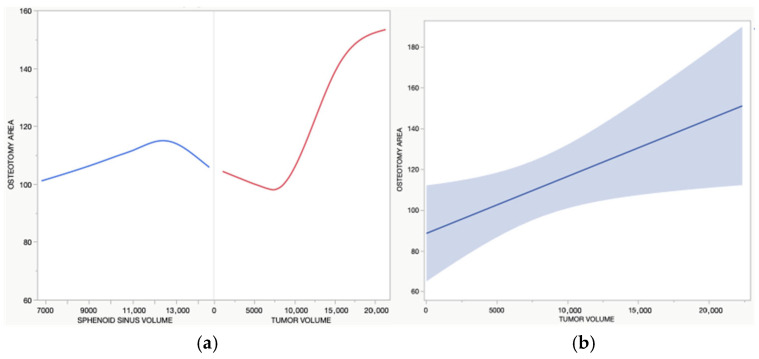
(**a**). The linear graph on the left illustrates the relationship between tumor volume (RED) and sphenoid sinus volume (BLUE), expressed in mm^3^, with the area of osteotomy expressed in mm^2^. (**b**). The graph on the right summarizes the model (blue line and area) of the relationship between tumor volume and the osteotomy area. The model has an R-square of 0.465 and a *p*-value of 0.0297. In summary, bigger tumors require a larger osteotomy for resection independent of other variables.

Postoperative imaging demonstrated moderate inter-patient variability in the osteotomy area within the cohort, with a mean of 109.67 mm^2^ (SD = 25.58 mm^2^). Exploratory multivariate linear regression identified tumor volume as the only variable significantly associated with the osteotomy area (R^2^ = 0.465, *p* = 0.0297). Larger tumors required larger osteotomies, independent of other factors such as Kassam grade, sphenoid sinus volume, intercarotid distance, age, or hypothalamic invasion (Figure 4).

### 3.2. Endocrinological Outcome

**Figure 5:**
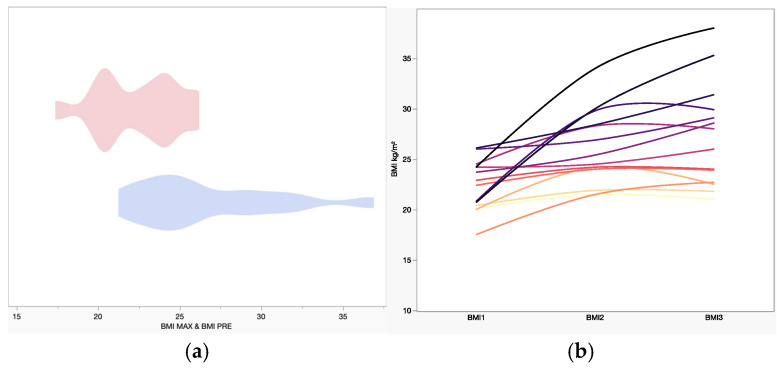
(**a**) The violin graph displays the preoperative BMI (RED) and the postoperative max BMI (BLUE). The mean preoperative BMI value is 22.39, while the maximum is 27.51. The statistical difference between the two groups is confirmed by a two-tailed *t*-test (*p* = 0.005). (**b**) This chart shows the trend of BMI (Body Mass Index, expressed in kg/m^2^) for each patient (in different colours), collected during the preparatory phase (BMI1), at the first follow-up (BMI2), and at the latest available follow-up (BMI3). During the follow-up there is an increase in the BMI values, statistically significant. In terms of the distribution of results, we can observe that, in many cases, there is a plateau in BMI increase, and in some cases, even a long-term reduction.

Diabetes insipidus was observed more frequently after surgery (Table 3, *p* = 0.0272) without a clear differential association with surgical approach. A significant postoperative increase in BMI was observed across the cohort (*p* = 0.0049). The mean preoperative BMI was 22.39 kg/m^2^ (range: 17.50–31.6), increasing to 26.65 kg/m^2^ (range: 21.5–36.7) postoperatively (Table 4). The most substantial BMI increase occurred in case 10 (+77.29%), while case 14 showed minimal change (+2.04%), remaining essentially stable (Figure 5). BMI changes did not show a clear association with the surgical route. Hypopituitarism increased after surgery (Table 3), without a clear differential pattern between endoscopic and transcranial approaches. Exploratory regression analyses did not demonstrate a clear association between surgical approach and endocrinological outcomes, including diabetes insipidus, hypopituitarism, or BMI increase.

## 4. Discussion

The optimal management of CPs remains debated [9,25,26], with no consensus regarding the preferred surgical approach, the extent of resection, or postoperative management strategy [26,27]. Despite excellent long-term survival rates, surgical treatment is frequently associated with substantial morbidity, particularly involving visual function and the hypothalamic–pituitary axis, with enduring consequences for quality of life [2,3,9,10]. Within this context, our study aimed to explore whether patient-specific skull base anatomy influences surgical approach selection and to examine long-term endocrinological outcomes in a contemporary adult cohort.

Surgical approach selection in CPs is inherently multifactorial, reflecting the interplay between tumor characteristics, skull base anatomy, surgeon experience, and institutional practice. Although transcranial approaches remain appropriate for lesions with marked lateral extension or a predominant intraventricular component, they have been associated with higher perioperative morbidity in several series [28]. Conversely, endoscopic endonasal approaches have gained increasing acceptance, supported by improved visualization of the sellar and suprasellar regions and potential advantages in optic apparatus decompression and stalk identification [27,29,30,31]. Nevertheless, available evidence, particularly in adult populations, does not consistently demonstrate superiority of one approach over the other, underscoring the need for individualized decision-making rather than algorithmic selection [30]. In our Center, an endoscopic endonasal approach was favored when tumor configuration and skull base anatomy were considered favorable, in accordance with established endoscopic classifications such as the Kassam one [24]. Key factors included a predominantly midline tumor location and an adequately developed sphenoid sinus, allowing a safe and effective transsphenoidal corridor. Conversely, a transcranial approach was preferentially selected in cases with marked lateral extension beyond the parasellar region, a predominant intraventricular component, or unfavorable sphenoid sinus anatomy limiting endonasal access. This individualized selection strategy should be considered when interpreting comparative outcomes, as it may have influenced the distribution of anatomical complexity between surgical groups.

In this framework, our findings highlight the relevance of skull base anatomy as a contributory factor in approach feasibility. Specifically, larger sphenoid sinus volumes were associated with selection of an endoscopic endonasal route. This observation is clinically intuitive: when the nasal phase is excluded, the transsphenoidal corridor is constrained primarily by the sphenoid and the sellar floor osteotomy [32]. Volumetric assessment captures these spatial relationships more comprehensively than linear measurements, supporting its use as an adjunct to surgical planning rather than as a stand-alone determinant. Importantly, these data are exploratory and should be interpreted within the broader clinical context and not as rigid anatomical thresholds.

The use of 3D modeling represents a methodological strength of this study. By integrating volumetric reconstructions of both tumor and skull base anatomy, this approach provides a more realistic appraisal of the operative corridor and its constraints. In line with this hypothesis, de Notaris et al. demonstrated the feasibility of generating high-fidelity 3D reconstructions of the sphenoid sinus and sellar region, capturing spatial constraints that are not fully appreciated with conventional 2D imaging. Although primarily proposed for educational purposes, these techniques reinforce the relevance of volumetric assessment of the surgical corridor, which is particularly critical in EAs [33]. Our experience suggests that patient-specific 3D modeling may facilitate surgical planning and multidisciplinary discussion, although reproducibility across centers and operators warrants further validation.

Among patients undergoing endoscopic surgery, the sellar floor osteotomy area, representing the narrowest segment of the transnasal corridor [27], was directly related to tumor volume and independent of sphenoid sinus size, intercarotid distance, or patient-specific anthropometric variables. This finding aligns with prior reports indicating that sphenoid sinus geometry alone does not dictate sellar exposure [14,34,35]. Rather, osteotomy size appears to be deliberately tailored to tumor burden, balancing adequate exposure against the risk of cerebrospinal fluid leakage and vascular injury [14,27,34,35]. When patients are appropriately selected for an endoscopic approach, anatomical variability does not appear to limit exposure or extent of resection.

With respect to oncological outcomes, gross total resection was achieved in the majority of cases, with subtotal resection reserved for lesions with significant hypothalamic adherence. This reflects contemporary practice, in which the absence of a clear surgical plane and the risk of hypothalamic injury represent the principal constraints to radical resection [36]. While gross total resection is associated with lower recurrence rates, subtotal resection followed by adjuvant radiotherapy remains a valid strategy in selected cases [36,37]. In our cohort, all patients undergoing subtotal resection received adjuvant radiosurgery, with only one recurrence observed during follow-up.

Endocrinological morbidity remains a major determinant of long-term outcome after CP surgery. Hypothalamic obesity, in particular, represents one of the most disabling sequelae, typically manifesting early after surgery and often stabilizing over time. The postoperative increase in body mass index observed in our cohort, together with the characteristic pattern of early gain followed by plateau [7], is consistent with hypothalamic involvement described in prior series [25]. These effects frequently require long-term, multidisciplinary management and may persist despite optimal surgical technique. In this series, endocrinological morbidity appeared more closely related to tumor anatomy and hypothalamic involvement than to the surgical corridor itself. The present analyses were observational in nature and were not designed to establish causal relationships between surgical technique and endocrine outcomes.

Diabetes insipidus was also a frequent postoperative finding, in line with the existing literature [30,38]. Its occurrence has been linked to both direct hypothalamic injury and vascular compromise, even in stalk-sparing resections, and does not consistently differ between endoscopic and transcranial approaches [5,37,38]. The close association between diabetes insipidus and hypothalamic obesity, particularly in high-grade tumors, further supports the concept that posterior hypothalamic involvement plays a central role in postoperative endocrine dysfunction [39]. Several studies propose that DI may serve as an early marker of HO, given their shared association with posterior hypothalamic injury [27,29,40]. We support this theory: the hypothalamus, particularly its posterior region, has a mean volume of ~25.93 mm^3^, while the mean tumor volume in our cohort was ~7768 mm^3^ [41]. Given the marked disparity between tumor volume and hypothalamic size, some degree of hypothalamic disturbance may be unavoidable in many cases.

This study has several strengths, including long-term follow-up with detailed endocrinological assessment and the application of volumetric skull base analysis, which remains underreported in the craniopharyngioma literature. The consistency of surgical technique, with all procedures performed by the same senior surgeon, further limits procedural heterogeneity. This is an exploratory study, and the retrospective design, small cohort size, and prolonged inclusion period should be considered when interpreting the findings. While it is not possible to entirely exclude a selection bias in the choice of surgical approach, potentially influenced by sphenoid sinus anatomy, these factors do not diminish the potential utility of our volumetric planning tool as an adjunct in preoperative assessment.

## 5. Conclusions

The management of craniopharyngiomas remains complex and requires individualized surgical planning. In this series, endoscopic endonasal and transcranial approaches resulted in similar outcomes, although this study was not powered to definitively establish equivalence. Quantitative assessment of skull base anatomy, particularly sphenoid sinus volume derived from 3D modeling, was associated with the selection of an endoscopic approach and may contribute to preoperative feasibility assessment. These findings support the role of patient-specific anatomical evaluation as an adjunct to surgical decision making rather than as a stand-alone determinant. Both gross total and subtotal resection strategies showed distinct risk–benefit profiles, underscoring the importance of balancing tumor control with hypothalamic preservation. Larger, prospective studies are warranted to further refine anatomy-informed surgical planning and its impact on long-term outcomes.

## Figures and Tables

**Table 1:** This table presents demographic data including sex, age (years old), total follow-up time (months), type of approach (TR: transcranial, ER: endoscopic), any previous procedures, subsequent major surgical procedures (surgical and non-surgical), and complications after major surgery. Recurrence, in this case, is considered a surgical complication.

	Sex	Age	Follow-up Time	Type of Surgery	Previous Procedures	Subsequent Procedures	After Surgery Complications
1	M	16	235	TR	Cyst stereotactic puncture, Ventricular shunt		
2	M	20	218	TR	Cyst stereotactic puncture	Ventricular shunt	Hemorrhage
3	F	49	72	ER			
4	F	67	97	ER		Radiosurgery	CSF leak
5	M	54	24	TR		Radiosurgery	Recurrence
6	M	63	62	ER			
7	M	45	37	TR	Ventricular shunt	Radiosurgery	
8	F	41	26	ER			CSF leak
9	F	46	18	ER			
10	F	51	23	ER			
11	M	29	13	ER			Infection without CSF leak
12	F	58	12	ER	Endoscopic cyst puncture		
13	F	47	12	ER			Infection without CSF leak
14	F	41	12	ER			CSF leak

**Table 2:** This table presents all the radiological and 3D model data. Tumor and sphenoid sinus volumes are expressed in mm^3^, inter-carotid distance in mm, and the osteotomy area in mm^2^. The extent of resection was evaluated on the early postoperative MRI: the presence of pathological residue was classified as STR (subtotal resection). In bold at the bottom, the mean values are reported.

	Kassam	Tumor Volume	Sphenoid Sinus Volume	Inter-Carotid Distance	Osteotomy Area	Extent of Resection
1	1	12,463	5424	14.94	-	GTR
2	1	1043	9989	16.98	-	GTR
3	3a	5408	11,086	14.18	87.38	GTR
4	3b	7562	10,845	18.58	76.64	STR
5	3a	5143	7926	13.63	-	STR
6	3a	6162	12,983	12.48	130.65	GTR
7	3b	13,887	10,300	11.42	-	STR
8	3b	8054	14,021	13.13	88.80	GTR
9	1	1429	9500	15.81	102.86	GTR
10	3a	15,218	12,318	15.52	149.70	GTR
11	3b	21,345	10,533	13.31	150.38	GTR
12	3b	3941	14,519	14.45	103.39	GTR
13	2	1047	10,870	14.79	105.31	GTR
14	3a	6055	6822	13.31	101.62	GTR
		**7768.36**	**10,509.71**	**14.47**	**109.67**	

**Table 3:** This table displays the clinical data about hypoadrenalism (H.adren), TSH deficiency (TSHdef), hypogonadism (H.gonad), and diabetes insipidus (ID). The absence of deficit is represented by “0”, while clinical evidence of deficit or diabetes insipidus is represented by “1”. The values are reported before the surgery (preoperative), before the discharge (early postoperative) and at the last follow-up available (last follow-up). The total values are reported in bold at the bottom.

	H.adren Preoperative	TSHdef Preoperative	H.gonad Preoperative	ID Preoperative	H.adren Early Postoperative	TSHdef Early Postoperative	H.gonad Early Postoperative	ID Early Postoperative	H.adren Last Follow-Up	TSHdef Last Follow-Up	H.gonad Last Follow-Up	ID Last Follow-Up
1	0	0	0	0	1	1	1	1	1	1	1	1
2	0	0	0	1	1	1	1	1	1	1	1	1
3	0	0	0	0	1	1	0	1	0	1	0	0
4	0	0	0	0	1	1	1	1	1	1	1	1
5	0	0	0	0	0	0	0	0	1	1	1	1
6	0	0	0	0	1	1	1	0	1	1	1	0
7	0	0	0	0	0	1	1	1	0	1	1	1
8	0	0	0	0	1	1	1	1	1	1	1	1
9	0	0	0	0	1	0	0	0	1	0	0	0
10	0	0	0	0	1	1	1	1	1	1	1	1
11	1	1	1	1	1	1	1	1	1	1	1	1
12	0	0	0	0	1	1	0	1	1	1	0	1
13	0	0	0	1	1	1	0	1	1	1	0	1
14	0	0	0	0	1	0	0	0	1	0	0	0
	**1**	**1**	**1**	**3**	**12**	**11**	**8**	**10**	**12**	**12**	**9**	**10**

**Table 4:** This table displays the clinical data about BMI. It is expressed in kg/m^2^, and values are reported at the time of surgery (BMI 1), at the intermediate evaluation (BMI 2) and at the last assessment (BMI 3). In the fourth column are reported the maximum value reached during follow-up (BMImax). BMI variation is calculated between the BMI1 and BMImax previously reported values. The mean values are reported in bold at the bottom.

	BMI 1	BMI 2	BMI 3	BMI Max	BMI Max Variation
1	17.50	21.50	22.70	22.70	29.71%
2	20.00	24.20	22.50	24.20	21.00%
3	22.40	24.00	23.90	24.00	7.14%
4	20.10	21.50	21.00	21.50	6.97%
5	22.90	24.20	24.00	24.20	5.68%
6	26.10	28.40	31.40	31.40	20.31%
7	23.70	25.40	28.60	28.60	20.68%
8	24.20	34.00	38.00	38.00	57.02%
9	24.20	24.50	26.00	26.00	7.44%
10	20.70	30.00	35.30	35.30	70.53%
11	26.00	26.90	29.10	29.10	11.92%
12	20.40	21.90	21.80	21.90	7.35%
13	20.80	29.80	29.90	29.90	43.75%
14	24.50	28.30	28.00	28.30	15.51%
	**22.39**	**26.04**	**27.30**	**27.51**	

## Data Availability

The data presented in this study are available upon request from the corresponding author. The data are not publicly available due to privacy and ethical restrictions.

## Abbreviations

The following abbreviations are used in this manuscript:

CP	Craniopharyngioma
HO	Hypothalamic obesity
DI	Diabetes insipidus
ROI	Region of interest
STL	Standard triangulation language
ER	Endoscopic resection
TR	Transcranial resection
MRI	Magnetic resonance imaging
CT	Computed tomography
STR	Subtotal resection
GTR	Gross total resection
ICD	Inter-carotid distance
IIIVCS	III ventriculostomy
EVD	External ventricular drainage
ITT	Insulin tolerance test
AUC	Area under the curve
